# Conventional and New Ways of Governing Forest Threats: A Study of Stakeholder Coherence in Sweden

**DOI:** 10.1007/s00267-017-0951-z

**Published:** 2017-11-02

**Authors:** Louise Eriksson

**Affiliations:** Department of Geography and Economic History, Umeå University, SE-901 87 Umeå, Sweden

**Keywords:** Forest risk governance, Stakeholders, Structural factors, Policy acceptability, Threat appraisals, Trust

## Abstract

Based on a framework for analyzing stakeholder coherence horizontally and vertically, the present study examined the governance of forest threats in Sweden. Opinions of forest risk governance in stakeholder groups with and without a connection to private forestry were compared (*n* = 2496) and the opinions were analyzed in relation to current governance practices. More specifically, forest threat appraisals, trust in the Swedish Forest Agency (SFA), and the acceptability of forest risk policy measures directed at private forest owners were assessed. Results revealed an overall coherence between different stakeholders in this context. However, the groups differed in, for example, the acceptability of the hypothetical regulative measure aiming to reduce damages threatening the forest long-term (e.g., climate change). Furthermore, an extensive use of advice for a fee may challenge particularly the internal, but also the external, legitimacy of forest risk governance. The forest owner stakeholder group showed lower threat appraisals when evaluating threat to one’s own forest rather than to the Swedish forest, except regarding browsing by animals. Regulations were not disapproved of in any of the stakeholder groups, although the forest owner group generally displayed higher acceptability of encouraging measures compared to the general public. Trust in the SFA was furthermore confirmed as an important driver of policy acceptability, and higher threat appraisals of novel threats, such as climate change and fire, resulted in a higher acceptability of measures less central or new in this context. The value of analyzing stakeholder coherence for natural resource management and governance is discussed.

## Introduction

Forests provide various ecosystem services, including provisioning, regulating, supporting, and cultural services (Millennium Ecosystem Assessment 2005). The health of forests is threatened by various stressors, however, including human (e.g., fire), climate (e.g., wind throw) and biotic ones (e.g., new or invasive pests and pathogens) (Trumbore et al. 2015). Even though some level of disturbance is desirable in a forest context, global climate change is expected to lead to an increase in damages, and new management strategies may be needed to avoid extensive damage (Fuhrer et al. 2006; Lindner et al. 2014; Seidl et al. 2014). Adaptation to climate change may involve active forest risk management, for instance planting more broadleaves and fewer coniferous trees, more mixed forests, and changing the rotation length and thinning schedule (Bouriaud et al. 2015; Fuhrer et al. 2006). The implementation of active forest risk management varies, however, and has even been portrayed as inadequate in many contexts (Blennow 2008; Flint et al. 2012; Lidskog and Sjödin 2014; Valente et al. 2015). To encourage forest risk management in countries with large numbers of private forest owners (e.g., the United States, Finland, Portugal, Germany, and Sweden), policy measures directed at the owners may be needed. Private forest policy measures may include, for example, regulations as well as informational and economic measures (Janota and Broussard 2008). With an increase in damages associated with a changing climate, forest risk governance may need to be intensified using additional and possibly stricter policies.

To avoid resistance and conflicts in natural resource management (e.g., erosion of relationships between actors, extreme activism), some level of coherence, or shared views, between different societal actors is important (Gritten 2009; Kozak et al. 2008). Forest interest groups vary greatly between countries and regions but may include, for example, forest owners, the forest industry and the responsible government, but also recreational, environmental, and indigenous groups (Berninger et al. 2009; Rantala and Primmer 2003). More broadly, however, any person, group or organization that has an interest in a matter may be labelled a stakeholder (Post et al. 2002). Since forests can be considered a natural common-pool resource they often have numerous beneficiaries albeit with different rights (e.g., bare access, right to extract from the resource, full ownership rights) (Ostrom et al. 1994; Schlager and Ostrom 1992). In many countries, forests are furthermore important national assets, and the general public may thus be considered a significant stakeholder in this context (Elasser 2007; Eriksson 2012).

At a time when forest management is being modified (e.g., as part of climate change adaptation), it is particularly important to consider the opinions of different stakeholders to make sure that management and governance are considered legitimate. Risk governance concerns the actions, institutions and processes involved when authority is being exercised in the risk domain, including the interplay with stakeholders (International Risk Governance Council 2012; Renn 2015). The present study examined stakeholder coherence with a focus on the governance of forest threats in Sweden, based on an analytical framework exploring intergroup correspondence (horizontally) and subjective legitimacy (vertically) (Eriksson 2012; Lundmark et al. 2014). The opinions of stakeholders with and without a connection to private forestry were compared and analyzed in relation to the policy measures currently in use in this context, but also to potential novel measures that may be used in the future.

### Framework for Analyzing Stakeholder Coherence in a Forest Context

**Fig. 1:**
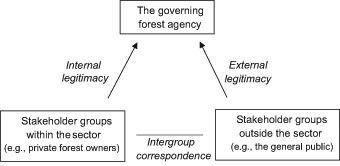
A vertical and a horizontal analysis to inform on stakeholder coherence in the forest sector

The extent to which there are shared views among stakeholders—that is, stakeholder coherence—was examined in this study through a horizontal and a vertical analysis (see Fig. 1). The horizontal analysis involves a comparison of the opinions of different stakeholder groups, revealing the potential for intergroup correspondence or conflict associated with a specific topic. More specifically, large deviations in the opinions of different stakeholder groups may be indicative of future forest conflicts (Eriksson 2012). In addition, large stakeholder groups may be comprised of several sub-groups. For example, shared experiences among people of the same gender may lead to gender differences in opinions (Gustafson 1998). Other structural factors besides stakeholder group (e.g., gender) may thus need to be considered to improve the understanding of opinions in this context.

The vertical analysis considering people’s opinions of the governing body and outcomes of governance is based on the concept of legitimacy, concerning the extent to which power is rightful (Lipset 1981). Subjective legitimacy refers to people’s perception of legitimacy (Hinsch 2010), and following Lundmark et al. (2014) it is possible to differentiate between internal and external legitimacy (see also Provan and Kenis 2007). Whereas internal legitimacy may be inferred based on the opinions of involved (or primary) stakeholders (e.g., forest owners in the forest sector), external legitimacy is based on the opinions of external (or secondary) stakeholders and the broader society (e.g., the general public). The analytical framework developed in this study aims to uncover stakeholder coherence horizontally and vertically, and to pinpoint concerns that are important for the design of future forest risk governance.

#### Opinions on forest risk governance

Attitude theory may be used to define and structure opinions on a specific topic. According to attitude theory, beliefs are cognitions, or thoughts, about an attitude object and salient beliefs are considered the building blocks of attitudes (i.e., a positive or negative evaluation of an attitude object) (Eagly and Chaiken 1993). To reveal opinions on forest risk governance, the present study focused on beliefs reflecting appraisals of forest threats and governance, but also attitude toward policy; that is, policy acceptability.

The perceived urgency of risk governance in stakeholder groups may be reflected in the subjective appraisals of a threat (or risk perception). Even though a more objective assessment of threats may play a role in subjective appraisals, there is no one-to-one relationship. Instead, people make their own assessment in terms of, for example, the perceived consequences of a threat, the probability of a threat, and evoked emotions (Reser and Swim 2011; Sjöberg 2000). In addition, people generally believe that they are less likely to be affected by a threat compared to others (i.e., general vs. personal threat appraisal), a tendency commonly labelled optimism bias (Becker et al. 2013; Sjöberg 2000). Opinions on governance may include beliefs of the governing body, such as social trust involving a willingness to rely on responsible actors, but also, for example, the decision processes in terms of procedural fairness (Siegrist et al. 2000; Tyler 2006). In addition, evaluations of various outcomes of governance in terms of decisions, policy goals and specific measures, etc., are relevant (Lundmark et al. 2014).

#### Horizontal analysis of forest risk governance

Previous studies have explored similarities and differences in the beliefs and attitudes held by different forest stakeholder groups (Berninger et al. 2009; Poudyal et al. 2015). For example, forest owners and the general public have been found to diverge in what they value in the forest (e.g., the importance attached to ecological values) (Eriksson 2012; Hellström 2001). However, studies comparing stakeholder groups’ opinions on forest risk governance are scarce.

For a horizontal analysis of opinions on forest risk governance, several structural factors may also play a role. In different contexts, women have been found to evaluate threats as more serious than men do, and gender (but also age) has been found to significantly predict threat appraisals with implications for management (Filipsson et al. 2014; Mumpower et al. 2016; Shavit et al. 2013; Slovic 1999). The influence of education on threat appraisals has been mixed, and whether more education will result in weaker or stronger concern regarding climate change is currently debated in the forest context (Blennow et al. 2016). Since subjective appraisals of threats may mirror the different levels of damage in a setting and the distance to the threat has been found to be relevant for appraisals (Sjöberg 2000; Uzzell 2000), structural factors associated with the place of residence may furthermore play a role in opinions on forest threats.

The present study draws on the cognitive hierarchy model to understand links between structural factors, beliefs and attitudes. According to a cognitive hierarchy model, structural factors, together with more general social psychological factors (e.g., worldviews, beliefs and cognitions), have been used to explain attitudes and behaviors in the environmental domain (Dietz et al. 1998) and with respect to forests specifically (McFarlane and Boxall 2003). Whereas this model suggests that structural factors may have a direct impact on attitudes, the effect may also be indirect, for example via beliefs.

#### Vertical analysis of forest risk governance

In previous research, vertical analyses of forest governance have been carried out. The subjective legitimacy of the forest governing agency has been found to be reasonably high within and outside the forest sector (Valkeapää and Karppinen 2013), also in the domain of forest risk governance (Toman et al. 2014; Vaske et al. 2007; Winter et al. 2004). However, results indicate that under some circumstances, or for certain issues, the evaluation of the governing body may be more negative. For example, a study from the US revealed a fairly negative evaluation of governing agencies in the public after the area had been affected by a Mountain Pine Beetle outbreak (Kooistra and Hall 2014), and low confidence in the ability of forest governing agencies to deal with climate change was evident in the public in Canada (Hajjar and Kozak 2015).

A vertical analysis may also focus more specifically on stakeholders’ evaluations of policy measures. Although not pertaining to risk governance, different stakeholder groups in the US have been found to be more supportive of economic incentives than of regulations used in the governance of private forests. However, whereas the public was close to neutral towards regulative measures, landowners with medium or large-sized forests were negative suggesting that the level of subjective legitimacy may differ between stakeholder groups (Poudyal et al. 2015; Schaaf and Broussard 2006).

### The Present Study

The present study analyzed stakeholder coherence horizontally and vertically in relation to the governance of forest threats in Sweden. For the horizontal analysis, the opinions of people with a connection to private forestry (either being a forest owner or belonging to a forest owning household) and the general public with no such connection were compared. Opinions on forest risk governance covered beliefs about forest threats and the governing agency, but also attitudes towards policy to cover key aspects of forest risk governance. More specifically, the analyses focused on: (1) threat appraisals of nine forest threats (with human and/or natural causes and varying in the frequency and intensity of occurrence in the past); (2) trust in the Swedish Forest Agency (SFA); and (3) private forest policy acceptability of six measures aiming to improve the owner’s risk management. The set of examined policy measures included regulations as well as informational and economic measures, representing both conventional and new measures in a Swedish context.

As part of the horizontal analysis, the role of stakeholder group was further examined in connection to other structural factors and beliefs. Because the stakeholder groups were large and heterogeneous, the importance of structural factors including gender, age, education, and place-based factors (i.e., region and urban/rural setting) for opinions was explored. Following the cognitive hierarchy proposed by Dietz et al. (1998), the importance of structural factors for beliefs (i.e., threat appraisals and trust in the SFA) was examined. In addition, structural factors and beliefs were examined as predictors of policy acceptability. Hence, structural factors were expected to be related to beliefs, and both structural factors and beliefs were presumed to be related to attitudes.

The vertical analysis was conducted by considering the level of trust in the SFA in the different stakeholder groups within and outside the forest sector, thus focusing on both the internal and external legitimacy of forest risk governance. In addition, the use of different policy measures in the Swedish context was related to the stakeholder groups’ acceptability of policies to assess coherence between governance practice and stakeholder opinion.

## Methods

### The Context

Forest covers nearly 70% of the land area in Sweden, and approximately half of the forest is owned by almost 330,000 private forest owners (also labelled individual family forest owners or non-industrial private forest owners) (SFA 2014b). Whereas storms have caused the greatest economic loss in forestry in Sweden, insects and fungi, as well as browsing damage, are also serious problems (Swedish Government Official Reports (SOU) 2007). With a changing climate, the forest damage caused by several of these threats is furthermore expected to increase in the future. Notably, though, there are geographic variations in damages and, for example, damage from storms and browsing animals is currently greater in the south compared to the north of Sweden (Blennow 2013; Skogseko 2014 [Official magazine published by the SFA]). In addition to natural threats, forest management practices such as clear-cutting may cause rutting, and the recreational activities of the general public (as a result of the extensive public access to forests in Sweden) may lead to problems of littering and heavily used paths (Eriksson 2014; SFA 2014a). Since 1993, the environmental and production objectives are equally important in the Swedish forest policy (Swedish Government Bill 2007/08:^108^). Information and advice are the most important instruments to attain the goals in the forest policy, although regulations and financial instruments are also used (Johansson and Keskitalo 2014). Forest owners are given a great degree of freedom in the policy and regulatory framework (the Swedish Forestry Act); although it is a “freedom under responsibility”, indicating a need to do more than the mandatory rules stipulate. The responsible agency, the SFA, is a national agency but with offices in more than a hundred different communities.

### Participants

The analyses were conducted based on data from two questionnaire studies. For one study, a sample of private forest owners (aged 20–80 years, owning more than 5 ha of forest land) was drawn randomly from the property register in Sweden (*n* = 3000). The response rate was 50% (*n* = 1482). For the other study, a stratified sample of residents (aged 20–75 years) was drawn from the Total Population Register in Sweden (from the counties of Skåne in the south, Västernorrland in the middle, and Norrbotten in the north) (*n* = 3000). The response rate was 34% (*n* = 1026). From these studies, three groups were extracted: private forest owners (*n* = 1482); and from the general public study, forest owning households (the respondent themselves or someone living in their household, e.g., a spouse owned forest) (*n* = 177) and the general public with no forest owner in the household (*n* = 837).^1^ In addition to enable a comparison between stakeholder groups, this set-up allowed for a comparison of how threats to one’s own forest vs. threats to the Swedish forest were evaluated by the forest owner stakeholder group.

### Measures

Measures for the questionnaires were developed based on theory and previous research. A forest damage expert at the SFA reviewed the selection of forest threats and policy measures. Before conducting the study, a few representatives from the respective target populations pre-tested the questionnaires. Only questions relevant to this analysis are described here. Questions about the respondent included gender, age, education, and place of residence. The variable region, corresponding to the organizational setup at the SFA, represented the home region of the respondents in the general public study, and the forest region in the forest owner study according to register data.

Appraisals of damage from different forest threats relevant in a Swedish context was assessed, including damage from: storms, insects (e.g., European spruce bark beetle), fungi (e.g., annosum causing root rot, pine twisting rust), browsing (e.g., moose), fire, the general public’s activities (e.g., during outdoor activities), climate change, new pests and pathogens (e.g., Dutch elm disease, ash dieback), and rutting caused by forest management. The measures incorporated the perceived probability of a threat together with the perceived consequences of a threat (cf. Sundblad et al 2007). Respondents in the forest owner study were asked the following question: “How likely do you believe it is that your forest would be impaired by the following within a time period of ten years?” (1 = Not at all likely, 5 = Very likely) (i.e., personal threat appraisals), and respondents in the general public study answered the question: “To what extent do you believe that the following damages constitute a threat to the Swedish forest within a time period of ten years (1 = Not at all, 5 = To a great extent) (i.e., general threat appraisals).

Trust in the SFA was examined by means of six items assessing value similarity (i.e., the SFA considers the needs of the public in their operations, the SFA have the same opinion about forests as I do), competence (i.e., the SFA completes its task in a suitable manner, the SFA knows enough to implement the goals in the forest policy [both environmental and production]), and trust (i.e., I lack trust in how the SFA manages forest threats (*R*), I have faith in how the SFA manages forest threats). The items were evaluated on a five-point Likert scale (1 = Totally disagree; 5 = Totally agree; Don’t know), and after reversing one item and excluding “Don’t know” answers, the means of the items were combined into an index variable with high reliability (Cronbach alpha, *α* = .84) (Vaske et al. 2007).

Questions on the acceptability of private forest policy measures were introduced by stating that the SFA can work in different ways to improve risk management in the forest. The respondents were then asked the following question: “Do you believe that the following are good or bad strategies for handling forest threats?: (1) Personal advice to forest owners for a fee; (2) Free advice to forest owners; (3) Regulations reducing the risk of short-term damage (e.g., removing storm-damaged timber to reduce the risk of insects); (4) Regulations reducing the risk of long-term damage (e.g., increased the demand for a more diverse forest); (5) Information to forest owners through meetings, excursions [in Swedish: *skogsträffar*] and courses; and (6) Economic subsidies to forest owners to improve risk management. Answers were given on a five-point bipolar scale (1 = Very bad, 2 = Rather bad, 3 = Neither bad nor good, 4 = Rather good, 5 = Very good). The selection of policy measures was guided by the literature on private forest policy (e.g., Poudyal et al. 2015; Schaaf and Broussard 2006) and the practice of forest risk governance in Sweden.

### Procedure

Statistics Sweden conducted the forest owner study in 2014 and the general public study in 2015 through postal questionnaires, including two reminders. The researcher was responsible for preparing the questionnaire and data analyses. SPSS Statistics 22 was used for analyzing the data. For the horizontal analysis (i.e., comparing the opinions of stakeholder groups), appraisals of the nine examined threats, trust in the SFA and policy acceptability were analyzed by means of univariate ANOVAs with stakeholder group as factor. Partial eta-square was used to evaluate the extent to which the stakeholder groups differed following the guideline proposed by Cohen (1988) (i.e., .01 = small difference, .06 = medium difference, .14 = large difference). To enable a comparison of personal and general threat appraisals, the three stakeholder groups were compared when analyzing threat appraisals, but for trust in the SFA and acceptability of policy measures, the two groups with a forest connection were collapsed into a single forest owner stakeholder group. For threat appraisals, partial eta-square was reported both for the full sample as well as separately for general threat appraisals (i.e., the general public (no forest owner) vs. the forest owning households) to assess the importance of stakeholder group for appraisals of general threat. To evaluate whether the appraisals of different forest threats may be considered a single measure, an exploratory factor analysis with varimax rotation was conducted on the nine threat appraisals.

Regression analyses were furthermore conducted to examine the importance of other structural factors than stakeholder group for threat appraisals and trust in the SFA. After gender, age, education, region, and size of place (see notes to Table 2) were dummy coded, the structural variables were included as independent variables and stakeholder group as control/s. When threat appraisals were analyzed, two dummy variables (distinguishing between the three groups) were included in the analyses, whereas in the analysis of trust in the SFA a dummy based on two groups (i.e., the general public (no forest owners) vs. the forest owner stakeholder group) was used. Another set of regression analyses was conducted to examine predictors of policy acceptability, with stakeholder group (two groups), structural factors, threat appraisals, and trust in the SFA as independent variables.

For the vertical analysis, the level of trust in the SFA in the different stakeholder groups was analyzed. In addition, official SFA sources and published studies were utilized to determine how the SFA has used different policy measures in forest governance more broadly and specifically in relation to forest risk governance. Even though the focus was on governance practices, expectations and plans in the policy were also outlined. This information was subsequently related to the acceptability ratings in the stakeholder groups.

## Results

### Sample Characteristics

The respondents in the different stakeholder groups are displayed in Table 1. In the general public study, the respondents were overall slightly older, and a higher share had a university degree compared to the populations in the different counties. As a result of the stratification on county in the general public study, the share of respondents from rural areas was much higher compared to the share in the Swedish population (where approx. 85% live in areas with more than 10,000 citizens). In the forest owner study, the mean age was higher and men were slightly overrepresented compared to the population of owners. These deviations, although minor, are further commented on in the Discussion section.

**Table 1:** Forest owners, forest owning households and the general public (no forest owners) by gender, age, education, region, and size of residence

		*n*	Gender		Age			Education	Region		Size of residence	
Study	Groups		Men	Women	-45 years	46–64 years	65 years-	University degree	North /middle	South	Less than 10,000	More than 10,000
			%	%	%	%	%	%	%	%	%	%
The forest owner study	Forest owners	1482	75.0	25.0	11.2	44.4	44.4	29.3	57.5	42.5	80.2	19.8
The general public study	Forest owning households	177	53.1	46.9	25.6	45.2	29.2	36.0	90.4	9.6	67.6	32.4
	The general public (no forest owners)	837	51.4	48.6	31.7	41.8	26.5	39.7	65.1	34.9	41.3	58.7

As expected, the two samples from the general public study were relatively similar, although the forest owning households more often lived in rural areas compared to those with no such link to private forestry (*p* < . 05). Larger differences were found between the samples from the two studies (i.e., the forest owner sample had a lower share of women, was older, fewer had a university degree, and a larger share lived in rural areas compared to the respondents from the general public study, *p* < .05). However, the samples largely reflect the different populations they represent.

### A horizontal Analysis of Stakeholder Groups

**Fig. 2:**
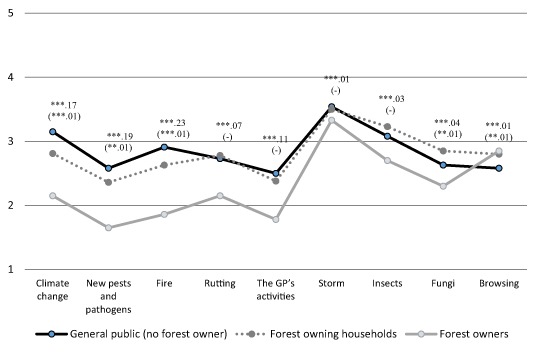
General threat appraisals (i.e., towards the Swedish forest) in the general public (no forest owners) and the forest owning households, and personal threat appraisals (i.e., towards own forest) among the forest owners (1 = low threat appraisals, 5 = high threat appraisals). Significance level (*p < .05, **p < .01, ***p < .001) and partial eta-square for the three stakeholder groups (and in brackets significance level and partial eta-square for general threat appraisals; - no significant difference)

Threat appraisals are displayed in Fig. 2 revealing that in general, threat appraisals ranged from low to moderate. The three groups agreed that storms were considered the greatest threat to the Swedish forest and the owners’ own forest, respectively. The main difference between the stakeholder groups was due to the distinction between general vs. personal threat since the forest owning households showed a higher concern for damages to the Swedish forest than the forest owners did for damages to their own forest, except in relation to browsing. However, forestry connection did influence the appraisal of some threats since the forest owning households perceived climate change, new pests and pathogens, and fire to be less serious threats to the Swedish forest, but fungi and browsing damage to be more serious, compared to those with no forestry connection in the general public.

An exploratory factor analysis of the threat appraisals revealed two factors with eigenvalues above 1 (4.040, 1.226) explaining 58.5% of the variance. Included in the first factor were damages caused by the general public, new pests and pathogens, fire, climate change, and rutting, while the second factor was comprised of damages by insects, storms, fungi, and browsing by animals. The threats in the second factor are thus threats causing greater damage to the forest in Sweden (according to more objective assessments) (SOU 2007). Hence, the first factor was labelled novel threats (*α* = .80) and the second common threats (*α* = .77).

**Fig. 3:**
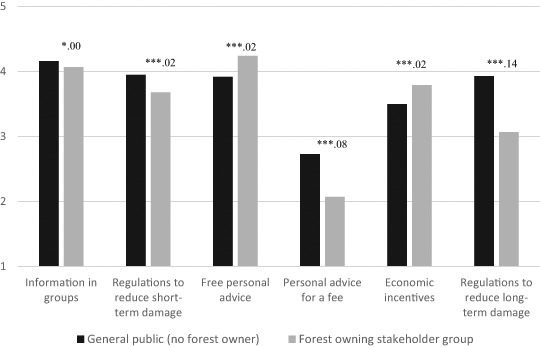
Acceptability of policy measures in the general public (no forest owner) and in the forest owning stakeholder group (1 = low acceptability, 5 = high acceptability). Significance level (*p < .05, **p < .01, ***p < .001) and partial eta-square for the two stakeholder groups

Trust in the SFA was slightly higher in the general public (*M* = 3.48, SD = 0.86) compared to the forest owner stakeholder group (*M* = 3.17, SD = 0.78) (*p* < .001, partial *η* ^2^ = .03) and group differences were evident in relation to all policy measures (see Fig. 3). Whereas the forest owner stakeholder group was more positive toward free advice and economic incentives, the general public was more positive toward regulations and advice for a fee. The most notable differences between the stakeholder groups were found in relation to regulations to reduce long-term damage, but also advice for a fee.

The regression analyses of threat appraisals and trust in the SFA revealed that, after controlling for stakeholder group, women were more concerned regarding both common and novel threats, and displayed higher trust in the SFA compared to men (see Table 2). Results further showed that respondents in the south displayed higher threat appraisals than those in the north/middle regions, and urban respondents displayed higher trust in the SFA compared to rural respondents. Notably, though, structural factors only explained a low level of variance in threat appraisals and trust in the SFA.

**Table 2:** Regression analyses for structural factors predicting threat appraisals and trust in the SFA (when controlling for stakeholder group)

	Common threats	Novel threats	Trust in the SFA
	β	β	β
Structural factors			
Gender (women)	.091***	.130***	.069**
Age			
Youngest	−.023	.014	−.009
Middle-aged	.032	.025	−.038
Education (university degree)	.036	.027	.011
Region (south)	.080***	.050**	.036
Size of place (urban)	−.019	.012	.054*
Control variables			
Stakeholder group (three groups)			
Forest owner study	−.078**	−.483***	–
Forest owning households (general public study)	.051*	−.029	–
Stakeholder group (two groups)			
Forest owner stakeholder group	–	–	−.128***
Adj *R* ^2^	.026	.279	.032

*Note*. Dummy coding of gender: women = 1, age (reference category oldest): youngest: 45 years or younger = 1, middle-aged: 46–64 years = 1, education: university degree = 1, region: south = 1, and size of place: urban representing more than 10,000 inhabitants = 1. Stakeholder group (three groups) (reference category general public no forest owners): forest owner study = 1, Forest owning households (general public study) = 1. Stakeholder group (two groups): forest owner stakeholder group = 1

*p < .05; **p < .01; * **p < .001

Results from the regression analyses examining predictors of policy acceptability are displayed in Table 3. Even after the inclusion of both structural factors and beliefs, the forest owner stakeholder group was more positive toward free advice and economic incentives and less positive toward regulations and advice for a fee compared to the general public. Furthermore, women displayed higher acceptability of information, free advice, economic incentives and regulations to reduce long-term damage. Younger respondents evaluated free advice and economic incentives more favorably than did respondents older than 65 years, and respondents with a university degree displayed a higher acceptability of information, advice for a fee and regulations to reduce long-term damage compared to their counterparts. In addition, respondents in the north/middle regions were more positive toward economic incentives compared to those in the south, and urban respondents evaluated advice for a fee more favorably than rural ones did.

**Table 3:** Regression analyses for structural factors, threat appraisals and trust in the SFA predicting policy acceptability

	Information in groups	Regulation to reduce short-term damage	Free personal advice	Personal advice for a fee	Economic incentives	Regulations to reduce long-term damage
	β	β	β	β	β	β
Stakeholder group (two groups) (forest owner stakeholder group)	.026	−.099***	.212***	−.184***	.237***	−.267***
Gender (women)	.065**	.036	.097***	−.034	.073***	.046*
Age						
Youngest	−.014	−.025	.097***	.037	.070**	−.009
Middle-aged	.040	−.016	.111***	−.010	.061*	.018
Education (university degree)	.091***	−.037	.024	.071**	.004	.048*
Region (south)	−.013	−.007	−.040	.004	−.053*	−.018
Size of place (urban)	.021	−.010	−.013	.065**	.003	.012
Common threats	.071**	.069**	.043	.008	.029	−.058*
Novel threats	.027	.010	−.002	.085**	.099***	.131***
Trust in the SFA	.316***	.240***	.230***	.113***	.172***	.217***
Adj R^2^	.131	.081	.097	.104	.072	.201

*Note*. Dummy coding of stakeholder group (two groups): forest owner stakeholder group = 1, gender: women = 1, age (reference category oldest): youngest: 45 years or younger = 1, middle-aged: 46–64 years = 1, education: university degree = 1, region: south = 1, and size of place: urban representing more than 10,000 inhabitants = 1

*p < .05; **p < .01; * **p < .001

Results furthermore revealed that trust in the SFA was important for the acceptability of all measures. However, whereas higher threat appraisal of common threats was associated with higher acceptability of information and regulation of short-term damage, it was related to a lower acceptability of the regulation of long-term damage. In contrast, higher threat appraisal of novel threats was related to a higher acceptability of advice for a fee, economic incentives, and the regulation of long-term damage. The levels of explained variance ranged from 7 to 20%.

### A Vertical Analysis of Governance and Stakeholder Groups

The level of trust in the SFA was at an intermediate level in both stakeholder groups, reflecting decent relations between the stakeholder groups and the governing agency (see A horizontal analysis of stakeholder groups). The SFA’s use of different forest governance measures is summarized in Table 4. Information and advice, given individually to forest owners, are considered primary measures in forest governance in Sweden, although the use of advice is changing (Eriksson 2017; Eriksson et al. 2010; Johansson and Keskitalo 2014). Whereas it has historically most often been given for free, in 1993 the forest policy stipulated that advice should be paid for as part of the commissioning work at the SFA. With external funding the provisioning of free advice has continued for some time, but the current lack of external funds has made it less frequent. At the same time, the use of advice for a fee has not increased much. Hence, in practice the role of advice is less important. Overall, the study supports a coherence between governance and the general public’s evaluation of policies, as the highest acceptability rating in the public was found for the principal policy measure information while in the forest owner stakeholder group both advice for free and information received very high acceptability ratings (Fig. 3). However, when it comes to advice for a fee, governance and opinions diverge as the aim in policy has been to increase the use of advice for a fee but this measure received low acceptability ratings in both stakeholder groups.

**Table 4:** Private forest policy measures in the practice of forest risk governance in Sweden

Information	Principal measure in forest governance, also in forest risk governance
Regulations to reduce short-term damage	As part of the framework law, a few but significant regulations in forest risk governance (e.g., removing storm-damaged timber to reduce risk of insects)
Free personal advice	High use historically in forest governance, but use is diminishing
Personal advice for a fee	According to the forest policy should be in more frequent use, but has not been widely implemented
Economic incentives	Marginal use in forest risk governance (e.g., encouraging specific management measures)
Regulations to reduce long-term damage	Has not been implemented as part of forest risk governance

Even though the Swedish Regulatory Act is a framework law, together with informational measures it has been described as an important tool for enacting the forest policy (Eriksson 2017; Johansson and Keskitalo 2014; SFA 2017). There are a few, but significant, mandatory rules with the aim of reducing the risk of short-term damage (e.g., the requirement to extract storm-damaged wood to avoid damage by insects), although regulations to reduce long-term damage (caused by, e.g., climate change) have not yet been implemented in this context. In this study, a relatively high acceptability in both stakeholder groups was revealed for the regulation of short-term damage. Whereas regulating long-term damage was also highly accepted by the general public, the forest owner stakeholder group was close to neutral. Although a few economic incentives are used in risk governance (e.g., using incentives to increase diversity to make the forest more resistant to damages), incentives may not be characterized as principal measures in this domain in Sweden (Eriksson et al. 2010). In the present study, the use of economic incentives was highly accepted by both stakeholder groups but the acceptability ratings were lower than for information and free advice.

## Discussion

Stakeholder coherence in the context of forest risk governance in Sweden was examined by comparing the opinions of different stakeholder groups horizontally as well as in a vertical analysis of opinions and governance practices. Both structural factors and beliefs were found to play a role for understanding the stakeholder groups’ opinions regarding forest risk governance. Furthermore, by pointing out exceptions to high stakeholder coherence, differences of opinion between the groups were identified and issues that may make governance less legitimate in the eyes of the stakeholder groups were highlighted.

The horizontal stakeholder comparison reveals high coherence between the general public and respondents with a connection to private forestry when it comes to, for example, appraisals of common forest threats (e.g., storms) and the use of information in forest risk governance. However, differences between the groups were more notable in appraisals of novel threats and acceptability of regulations to reduce long-term damage. The key difference in threat appraisals between the stakeholder groups can be accredited to the distinction between appraisals of threat to the Swedish forest vs. one’s own forest. However, even those with a connection to forestry evaluating threat to the Swedish forest perceived climate change, new pests and pathogens, as well as fire, as less serious compared to those external to the forest sector. The forest owner stakeholder group also displayed lower acceptability of the regulation of long-term damage. Hypothetical measures such as this one may be difficult to assess, but since it was not the least accepted measure, lack of experience is likely not the only reason for the lower level of acceptance. Results imply a somewhat lower readiness to deal with certain novel threats, and a lower acceptability of novel regulative (though not encouraging) measures within the forest sector compared to externally. Quite expectedly, the forest owner stakeholder group displayed lower acceptability of the regulative measures and advice for a fee but higher acceptability of the encouraging measures, including free advice, than did the general public. However, in contrast to studies from the US, where acceptability has been found to be higher for encouraging than regulative measures (Poudyal et al. 2015; Schaaf and Broussard 2006), the stakeholder groups in the present study were positive toward using different types of measures as part of forest risk governance, thus reflecting a broad readiness among different stakeholder groups for forest risk governance, despite a moderate or low concern regarding forest threats.

Among the structural factors, the stakeholder group was the most consistent predictor of opinions on forest risk governance. However, in line with studies of risk and risk management in other contexts (Slovic 1999), women generally displayed a more positive view of forest risk governance than men did. The present study revealed only minor differences in acceptability between respondents in different places (i.e., urban vs. rural and in different regions). Since place-based factors may be more important in other settings, however, and large differences may point toward intergroup divergence between people in different places, these factors are nevertheless relevant in a horizontal analysis of stakeholder groups. Higher explained variances in the models of acceptability highlight the importance of also considering the role of beliefs for opinions of forest risk governance. Adding to previous research on forest risk management (Vaske et al. 2007), this study confirms the importance of trust for the acceptability of forest risk governance measures. Trust in the governing agency was slightly more important for the acceptability of informational and regulative measures, but somewhat less important for evaluations of the economic measures, advice for a fee and economic incentives. The study further revealed a difference in the predictors of acceptability of principal vs. less central and novel measures in forest risk governance in Sweden. Whereas concern over common threats increased the acceptability of information and the regulation of short-term damage, the acceptability of also using measures that people are likely to have less experience with, including advice for a fee, economic incentives, and the regulation of long-term damage, was connected to a concern regarding novel threats. While free advice has been a principal measure in Sweden its use is diminishing, and acceptability of this measure was not significantly determined by any of the threat appraisals. Overall, an increase in the level of trust, but also more concern over novel rather than common forest threats, may increase the acceptability of a broader set of measures not in frequent use today.

Comparable to other contexts (Toman et al. 2014; Vaske et al. 2007), trust in the SFA was at an intermediate level in both stakeholder groups, and the relations between the governing agency and different stakeholder groups may thus be described as reasonable. The vertical analysis of policy acceptability and the use of policy further reveals that there was a fairly high level of coherence between the stakeholders’ opinions and the governance of forest threats in Sweden, with high acceptability for most measures used by the SFA. However, this analysis points to one issue of concern. A more extensive use of advice for a fee may reduce particularly the internal legitimacy of forest risk governance. Even though the negative evaluation of this measure among forest owners in Sweden could be a reaction to the need to start paying for something that has traditionally been provided for free, the public was not very positive either. The response may rather stem from a belief that the government should distribute knowledge free of charge.

Results suggest that the forest owner stakeholder group generally perceived threats to one’s own forest to be less serious than threats to the Swedish forest. Even though the slightly different response scales used for assessing general vs. personal threat may have contributed to this result, the difference was absent in relation to browsing and very minor for storm. Since the owners have had more experience of storm and browsing than of the other threats^2^, results indicate that the owners generally de-emphasize the seriousness of threats to their own forest but make a more realistic assessment of threats they have more experience of. Storms were perceived to be among the most serious forest threats, thus revealing some degree of overlap between more objective assessments of risks and subjective appraisals (SOU 2007). In line with this reasoning, the higher threat appraisals reported in the south compared to the middle and north of Sweden seem to be associated with geographic variation in damages. Notably, though, official documents suggest that, for example, fungi and browsing also cause serious damage to the Swedish forest, and these threats were perceived to be less serious in the present study (although those with a connection to private forestry displayed a slightly higher concern). The role of large-scale events in relation to threat appraisals is furthermore ambiguous. For example, whereas the large hurricanes that have damaged Swedish forest in recent years may have increased salience for storm damage (SFA 2014b; Witzell et al. 2009), the large forest fire in Västmanland in 2014 (just before the survey of forest owners) (SFA 2014c) does not seem to have had any great impact on appraisals. Since it is not only personal experience with damages and media reports, but also values and beliefs, that play a role in threat appraisals (Kasperson 1988; Slovic 1999; Weinstein 1989), the comparatively high concern regarding climate change damages in the general public revealed in this study may reflect a higher environmental awareness found in the general public compared to forest owners (Eriksson 2012). Overall, the understanding of threat appraisals requires the consideration of physical and societal, as well as individual, factors.

The present study used a random selection and a stratified random selection, respectively, to gather representative samples from the stakeholder groups, but certain deviations between samples and populations were evident, for example in age. However, since differences were minor and the importance of structural factors was examined in this study, these deviations were not expected to seriously threaten the validity of the main results. It is however important to note that since analyses were based on correlational data, causality between variables cannot be proved. Even though the data do not suggest that conducting the studies a year apart had any great impact on the results, this time lag needs to be considered when interpreting the results since events occurring in-between studies may influence opinions. Whereas previous studies comparing stakeholder groups have generally examined interest groups and thus used smaller samples (Berninger et al. 2009), the large samples in this study made it possible to provide a broad overview and consider structural differences based on socio-demographics in these heterogeneous stakeholder groups. Whereas acceptability helps to uncover the subjective legitimacy of governance, and may be considered one determinant of policy impact (cf. Gärling and Loukopoulos 2007), future studies should also examine to what extent the policy measures are effective in encouraging forest risk management among forest owners.

## Conclusions

With a changing climate, the emphasis on forest risk management is increasing (Landmann et al. 2015). From a managerial perspective, it has become even more important to consider the interconnectedness of different forest threats. This study contributes to a shift in how social science has approached forest risk management and governance, from a focus on only one type of forest threat (e.g., fire or insects) to a more comprehensive approach focusing on a range of different but sometimes related threats. When management and governance need to be transformed, stakeholder opinions are particularly relevant to consider. Although this study reveals a generally high stakeholder coherence within forest risk governance in Sweden, the key to legitimate forest governance lies in balancing the opinions external to the forest sector with internal opinions, for example when considering whether a broader set of measures is needed to deal with new forest threats. Since building trust is important in this context, it is imperative to have appropriate institutional structures and procedures in place, and make sure that the institution is highly qualified and able to connect with different stakeholder groups (Eriksson 2017; Stern and Baird 2015; Zucker 1986).

The framework developed in this study may furthermore be used to guide the analysis of stakeholder coherence in other natural resource management settings, considering governmental agencies on different levels (national, regional and local) as well as stakeholder groups directly involved in management but also the broader society. Disclosing different stakeholder groups’ opinions regarding current, but also potentially new, approaches in natural resource management and governance enables different interests to be considered during planning and implementation.

## Footnotes

1 In the general public study, 14 respondents had not indicated whether someone in the household owned forest and were thus excluded from the analyses.

2 A question in the forest owner study assessing experience of different damages revealed that the forest owners have had the most experience of storm (*M* = 2.49, SD = 1.14), followed by browsing (*M* = 2.25, SD = 1.02), rutting (1.73, SD = 0.78) and insects (*M* = 1.71, SD = 0.82) (Scale: 1 = Not at all, 5 = To a great extent).
